# A Review of Gait Phase Detection Algorithms for Lower Limb Prostheses

**DOI:** 10.3390/s20143972

**Published:** 2020-07-17

**Authors:** Huong Thi Thu Vu, Dianbiao Dong, Hoang-Long Cao, Tom Verstraten, Dirk Lefeber, Bram Vanderborght, Joost Geeroms

**Affiliations:** 1 Robotics & Multibody Mechanics Research Group (R & MM), Vrije Universiteit Brussel and Flanders Make, 1050 Brussels, Belgium; Dianbiao.Dong@vub.be (D.D.); Hoang.Long.Cao@vub.be (H.-L.C.); Tom.Verstraten@vub.be (T.V.); dlefeber@vub.ac.be (D.L.); bram.vanderborght@vub.ac.be (B.V.); jgeeroms@vub.ac.be (J.G.); 2 Faculty of Electronics Engineering Technology, Hanoi University of Industry, Hanoi 100000, Vietnam; 3 School of Mechanical Engineering, Northwestern Polytechnical University, Xi’an 710072, China; 4 College of Engineering Technology, Can Tho University, Can Tho 90000, Vietnam

**Keywords:** gait phase detection, event detection, lower limb prosthesis, gait phase classification, IMU sensor, smart insole, wearable sensors, assistive devices

## Abstract

Fast and accurate gait phase detection is essential to achieve effective powered lower-limb prostheses and exoskeletons. As the versatility but also the complexity of these robotic devices increases, the research on how to make gait detection algorithms more performant and their sensing devices smaller and more wearable gains interest. A functional gait detection algorithm will improve the precision, stability, and safety of prostheses, and other rehabilitation devices. In the past years the state-of-the-art has advanced significantly in terms of sensors, signal processing, and gait detection algorithms. In this review, we investigate studies and developments in the field of gait event detection methods, more precisely applied to prosthetic devices. We compared advantages and limitations between all the proposed methods and extracted the relevant questions and recommendations about gait detection methods for future developments.

## 1. Introduction

**Figure 1:**
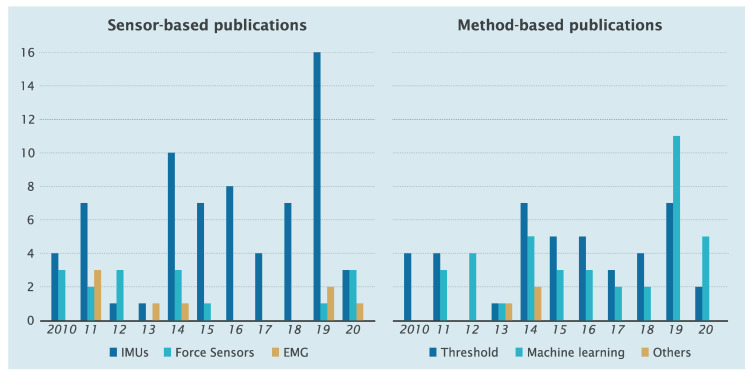
Number of sensor-based and method-based publications from 2010 to 2020.

Walking is a common and essential activity in most humans’ daily life. It is a complex process combining the functionalities of a variety of muscles and tendons. This optimized structure allows people to keep their balance, maintain stability, and move their body from one place to another. Even though walking is easy and straight-forward for able-bodied, it can be very challenging for specific groups of people to perform basic motions, not in the least for people who underwent lower-limb amputation due to sickness or trauma. Currently, technological advances in prosthetic design are trying to improve functionality through inventive mechanisms and powerful motors [1,2,3,4,5]. However, these devices can only provide good performance when paired with a functional gait phase detection algorithm. These active prostheses aim for increased safety, smooth motion, and comfort for the amputee. In the control system of the prostheses, the main controller needs the gait phase detection unit to provide information about the current gait phase. After detecting the current phase, the prosthesis can produce the corresponding desired ankle functionality. Therefore, adding an accurate gait detection strategy in the prostheses can improve the performance of the control system. For instance, the Ankle Mimicking Prosthetic Foot (AMP-Foot) has been developed to restore the loss function of below-knee amputees [6]. The gait detection was used to provide the required parameters for controlling the torque of the motor. During the second phase of gait from Foot Flat (FF) to Heel Off (HO), the AMP-Foot stores energy in the springs, then releases this stored energy, and transmits it to the ankle joint by controlling the DC motor at the moment of HO. This joint effort produces a peak torque as the power output then generates a Toe Off (TO) event. After the moment of TO, the prosthesis enters a swing phase when the motor torque is downward to a zero magnitude allowing the amputee to move to the next Initial Contact (IC) of the same foot. Based on the AMP-Foot working processing, at least three events including IC, HO, and TO need to be detected in order to precisely control the prosthesis. The number of required phases and events depends on the requirements of the prosthetic design. For different levels of granularity, from two to eight phases, the names of phases and events were standardized [7] and shown in Figure 1. The number of detectable phases and events depends on the methods and sensor systems adopted to gather the signal for recognition of the phases and events.

Numerous valuable methods of event and phase detection were presented. These computation methods are categorized into two main domains. Firstly, the domain based on the threshold method [8,9,10,11,12,13,14,15,16,17], time-frequency analysis [18,19,20,21], and peak heuristic algorithms [16,19,22,23,24,25], which are also variations of the threshold method. Secondly, Machine Learning (ML) approaches are now among the most popular techniques to detect phases and events with various models such as Hidden Markov Models (HMM) [26,27,28,29,30,31,32,33,34], or several of the latest studies published based on the Artificial Neural Network technique (ANN) [35,36,37,38], Deep Learning Neural Network (DLNN) [39,40,41,42,43], a Convolutional Neural Network (CNN) [44,45,46], or [28] proposed a hybrid method that combined HMM and Fully connected Neural Networks (FNN). Different computation methodologies provide different performances regarding the parameters such as the number of detectable phases, events, and detection delay, which will be discussed in the next section.

The models are adopted depending on the type of sensors that are installed for recording the signals of the gait. Nowadays, wearable sensors are widely used for gait phase recognition systems: Wearable force-based measurements [9,21,26,47,48,49,50,51,52,53,54], Electromyographic (EMG) sensors [55,56], Inertial Measurement Units (IMUs) [9,19,29,41,57,58], and joint angular sensors [24,59,60,61,62] are used specifically for the detection of the gait. The studies showed that the methods that used force-based measurements such as Force Sensing Resistors (FSRs), footswitches, and foot pressure insoles yield the highest precision for detection [7]. Nevertheless, the accuracy and reliability of the model depend on the sensor position under the foot. The highly sensitive force signals have to be processed to remove the noise, which will cause the detection delay. Additionally, the lifespan of the force sensor is primarily dependent on the mechanical wear. They are not durable as a consequence of being exposed to shock forces by the repetitive impact of the foot to the ground. Therefore, force sensor measurements are not the best solution for real applications and they are often applied for indoor experiments as the reference of the gait recognition. These days, the use of EMG sensors, which measures the activity of specific muscles during movement is less common than other wearable sensor systems as the complex requirements in data acquisition and processing steps [56]. Additionally, these sensors are really sensitive to the moisture that builds between the skin and sensors, and to how they are placed on the skin, concerning the muscles underneath. Recently, IMUs consisting of a combination of gyroscopes, accelerometers, and magnetometers have become widely utilized. They are portable, cheap, durable, reliable, consume low energy, and can easily be mounted on different body parts. These sensors also provide rich signal information about the gait angular velocity and acceleration that can be used for an accurate prediction of the gait events and phases, which is the main reason why IMU signals are a good option to use in a machine learning approach. In this paper, we review the advancements of algorithms for gait phase detection using wearable sensors, applied to the lower limb prosthesis. We evaluate the advantages and disadvantages of each system based on the specific requirements for different methods.

This review is organized as follows: Section 2 describes the methodology of the review. Section 3 depicts the overview of human walking gait granularity division. The details of human gait methods of using wearable sensors are analyzed in Section 4. A comparison of the performance between methods of existing studies are shown in Section 5. Finally, conclusions are stated in the last section.

## 2. Methodology

### 2.1. Search Strategy

In order to obtain related publications, a collection of studies were searched through the main databases i.e. Scopus, ScienceDirect, and Google Scholar concerning gait detection topics. Keywords used in the searching process are combinations of gait phase detection, gait event detection, recognition, classification, and wearable sensors.

The inclusion criteria for these searches were evaluated using the titles and abstracts with the following criteria. Manuscripts are written in English and have been published from January 2010 to June 2020. We focused on applications in medicine, rehabilitation, assistance, and research. We searched for conference and journal publications that explicitly presented gait detection for devices. Books, book sections, and review articles were excluded. The electronic searching initially found 340 publications in this period related to the concern topics. In a second screening based on the abstract, duplicates and out-of-scope papers were removed. Finally, based on a full-text screening, 84 articles that showed the outcome defined and measurable were retained.

### 2.2. Search Quantity

In this review, we discuss these criteria revolving sensor types, sensor placement, the performance of gait phase, and event detection methods based on several parameters such as accuracy, delay, and granularity of the cycle phases, as well as events detected. We utilize the following benchmark to examine the reviewed publications.

- Are the addition/rejection principles described and relevant to the prosthetic design?
- Is the outcome defined and measurable?
- Are essential baseline variables scaled, valid, and reliable?
- Is the data collection method identified and appropriate?

The overview considers research from 2010 to June 2020. We found 87 articles that concerned the gait phase or event detection using wearable sensors. Figure 1 indicates the number of published papers based on wearable sensors that measure the signals and methods applied:

- *Wearable sensors*: IMU sensors were the most commonly used (67 articles), much more than force sensors (12 articles) and EMGs (7 articles);
- *Methods*: The number of published threshold-based method articles (44) were nearly equal machine learning-based method articles (40), and other methods of 3 papers [53,63,64]. Though applications of Machine Learning-based have been increasing over the years, thresholding-based research studies were used more than other methods. This is because the computation of the thresholding method is simple and easy to apply in real-time.

A review of gait phase detection algorithms for lowerlimb prostheses.

## 3. Overview of Human Walking Gait Phases

A full gait cycle is defined as a periodic cycle involving two legs from the IC of one foot on the ground to the following occurrence of the same event with the same foot [41]. Typically, a gait cycle is divided into two main phases. The *stance phase*, which represents approximately 60% of a gait cycle, starts when the foot strikes the ground and ends when it leaves the ground. The *swing phase*, which accounts for approximately 40% of the remaining gait cycle, starts when one foot leaves the ground and lasts until it touches the ground again [65]. The IC marks the beginning of the stance phase and the TO marks the beginning of the swing phase. A normal walking gait cycle exhibits two single limb supports and two double limb supports. Double limb support is defined as the period when both feet are on the ground, whereas single limb support is the time when just one limb is on the ground.

**Figure 2:**
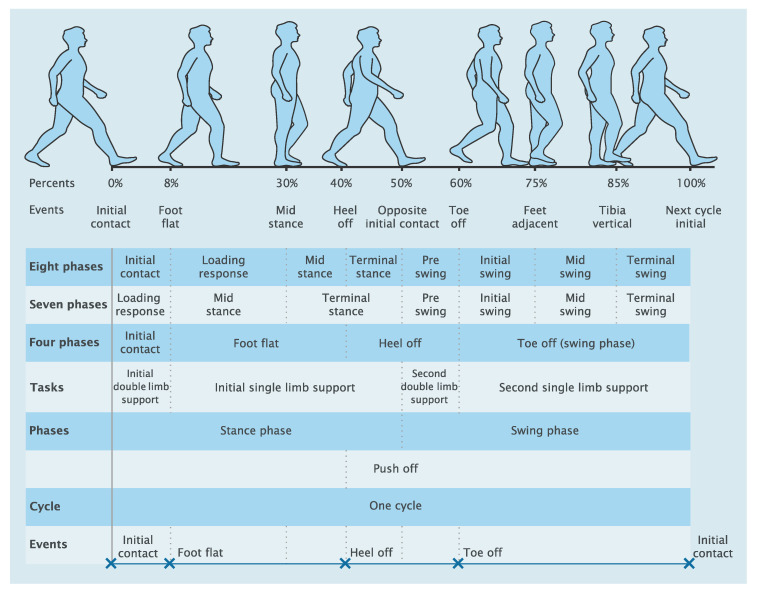
The fundamental division of a gait cycle. Figure adapted from [66].

Different gait terminologies are used in different works. In this paper, we chose to follow the wording of Figure 2. In this figure, a gait cycle percentage is defined as a sample from the continuous space of the gait cycle; an event is viewed as a discrete representation of the percentage space, often labeled as IC, FF, HO, and TO; a gait period is considered an interval between events; and a gait phase is considered a union of several periods that represent different stages of the gait cycle. Figure 2 illustrates eight periods of the gait cycle and synthesizes the names of the gait events and phases [7,65].

Regarding the gait fundamentals, a dynamic continuous occurrence of phases during one cycle is described from the heel strike at 0% to the next heel strike at 100% of the gait cycle. At the first period, 10% is *loading response* and the body weight is transferred onto the limb. Then the next period from 10% to approximately 30% is *mid-stance*, which is the first half of the single limb support period. The following 10% of the gait is the *terminal-stance*. Toe off or propulsion phase occurs after the foot-flat from 40% of the gait. The transition between stance and swing is *pre-swing*, which extends from 50–60% of the gait. The body is pushed to move ahead and prepare to push off the foot to enter the swing phase from roughly 60% of the gait. Single limb support appears from foot flat until 50% of the cycle is related to the opposite initial contact limb. The second double limb support occurs during the period between 50% until the toe leaves the ground at 60% of the step. Then the second single limb support starts until finishing the cycle. The actual swing is divided into three phases: *Initial swing* is early swing at approximately 60–75% of the gait cycle and *mid-swing* phase at approximately 75–85% of the gait cycle. The last period *terminal-swing* is between approximately 85% and 100% of the gait cycle. The fundamentals of human gait phases are shown in Figure 2.

## 4. Gait Detection Methods

Almost all methods in existing studies used the gait data of able-bodied subjects applying for the gait phase detection systems. For this reason there is the distinction between gait phase estimation based on only the amputated leg or also the sound leg, as the sound leg will always be closer to a healthy human gait [67]. But, in the end, both legs will be different from the healthy gait, so this also needs to be taken into account for example when using healthy gait data to train algorithms. Timings for example differ from what is described in the "healthy" human gait division, as amputees’ stance phase on the healthy leg will be usually longer than the one of the prosthetic leg. The gait also could change significantly with a different type of prosthesis, which can also affect the outcome [68].

### 4.1. Wearable Sensors for Gait Detection

The development of more robust, more efficient, and smaller sensors in the last decade has allowed a large number of features and characteristics of gait to be analyzed and evaluated quantitatively and objectively. This enabled improvements in the performance of the gait detection systems. Practically, using wearable sensors has become more convenient to measure features of a human’s gait. This section overviews different measurement sensors and their positions commonly applied for gait detection systems in gait rehabilitation and transtibial prostheses.

#### 4.1.1. Measurements Based on Force Sensors

The force sensor measurement method is to use force sensors placed on the sole under the foot to measure the contact between the foot and ground. Force sensors are often foot switches such as a simple force sensor based binary switches or FSRs, and foot pressure insoles.

Foot switches and foot pressure insoles have similar benefits and limitations of algorithms in the detection of gait phases since they have the same principles. They are the most effective methods to detect the stance phase and swing phase of the step and have simple signal inputs and high precision. Besides, force sensors are often exposed to shock forces when walking, hence they are durable, but also expensive.

In several works, these sensors have been implemented in applications [21,26,34,47,48,49,50,51,52,54,69,70]. First, Bae et al. [26] presented an analysis method based on HMM, which identified six gait phases using the outputs of four ground reaction forces. The method could also distinguish between abnormal and normal gait phases. However, the authors did not compute the performance of their method. Later, Agostini et al. [47] segmented four gait events by the use of data outputs from three foot switches mounted on the heel, the first metatarsus, and the fifth metatarsus. The method is based on a simple digital rule method, and was tested on both healthy and patient subjects, obtaining a reliable result of 100% accuracy in able-bodied subjects and 98% in disabilities. Cherelle et al. [51] presented the use of a FSR to accurately detect gait events of their prosthesis. However, these FSRs were found to be endurable, prone to mechanical failure, and aesthetically unpleasing. Moreover, they did not provide any information about sub-phases. Another method applied the multiple-regression HMM to recognize the six gait phases of an in-shoe pressure. This model was trained and unsupervised to avoid labeling manual data, especially, for training a large amount of data. Their proposed approach obtained an average recognition accuracy of 83.21%. Feng et al. [52] proposed a method to distinguish the stance phase and swing phase using one strain gauge bridge sensor instead of the load cell sensor to sense the contact between the carbon-fiber of the prosthesis and ground. The accuracy of this system was approximately 100%. However, this sensor only detected two phases as stance phase and swing phase, and could not obtain all possible granularities. Another study by Martini et al. [70] presented a gait event recognition making use of a simplified prototype of the pressure sensitive insoles based on optoelectronic sensors. The system was able to detect IC and TO events with a detection latency of 60 ms and 40 ms respectively, and an estimation stride error of 1.3%. Jiang et al. [54] explored a new phase classification technique of four phases based on a specific pressure pattern of Force Myography (FMG) embedded in an ankle band. The LDA method was employed to achieve a detection accuracy of 99.9% of all studied speeds. However, this method was considered for applications with slow walking speeds.

Due to their limited lifespan and because they are sensitive to the attachment of the insole, force sensors are not considered suitable for daily life applications. In spite of this, they are often used as the training label data [38,41,71] and a reference for validations [9,11,21].

#### 4.1.2. Measurements Based on IMU Signals

The most effective methods to measure the gait phases and events use IMUs. Standard IMUs measure the rotational velocity around three axis using gyroscopes, linear accelerations, and the strength of the magnetic field along those same axes, utilizing accelerometers and magnetometers. The methods based on measurement of the IMU signals present more advantages than the sensors mentioned. They are portable, low energy consumption, low cost, durable, and reliable. IMUs are often attached to the different body parts of the foot, the shank, or the thigh in order to capture the motion signals. IMUs support three types of signals in three axes as angle velocity, acceleration, and magnetism. However, several gait phase classification methods have been developed using the measurement of acceleration data [12,72,73,74,75,76] or gyroscope data alone [11,22,27,28,30,32,71,77,78,79,80]. The use of whole signals of IMUs, both angular velocity data and acceleration data, has risen recently to be more robust to detect the gait phases and events [9,21,41,81]. Various sensor positions of IMUs were tested as Mo et al. [72] determined the peaks of acceleration signals from three IMUs mounted on to the foot, pelvis, and shank to detect HC and TO events. The most accurate of IC detection was by MAD (Mean Absolute Difference) with 4.7±4.1 ms of their methods. Rueterbories et al. [12] proposed a thresholding-based algorithm to recognize phases from acceleration signals placed on the foot. The method could classify four phases with an accuracy of 84.2% with able-bodied subjects and 108.5% with patients. Another new approach method called Long Short-Term Memory-Deep Neural Network (LSTM-DNN) was introduced by [73]. This study used acceleration signals from three IMUs attached to different body parts: Foot, calf, and thigh to detect two main phases offline. This algorithm was tested on different test subjects and walking speeds, and the result reported that this gait-phase recognition accuracy was higher than 91.8%.

These papers showed that the use of accelerometers could be placed on different body segments such as the foot, shank, thigh, or pelvis involved in the gait. The data of acceleration showed specific peaks at the start and end of the stance phase and two events could be detected based on the threshold method or the heuristic peaks [12,72]. Hence, the accuracy of using accelerometers with the methods mentioned above showed poorly in regard to specific applications. Different from accelerometers, the use of gyroscopes reported greater results and various papers proposed applications using them [11,22,27,28,30,32,71,77,78,79,80]. In order to record the angular velocity of the joints from gyroscopes, IMUs were mounted in different positions: The foot, shank, thigh, and hip. The studies showed the sensor positions on the foot and shank obtained a higher classification accuracy with the sensor positions on the thigh and hip. Taborri et al. [71] showed the values of Min Time (>0.98) for the classifier with the angular rate signal of the foot, and the acceptable values of that (>0.95) for the classifier when the angular rate of the shank and thigh were processed. Various studies showed the angular velocity of the joint was the greatest suited HMM approach because of the reliable detection accuracy and detected four phases. [27,30,32,71,80]. Mannini et al. [30] presented HMM for the detection of four gait phases HS, FF, HO, and TO online using one IMU placed on the foot to measure the angular velocity in the sagittal plane. The results presented on overage of 45 ms (FF) early detection to 35 ms of late detection (HO). Their system [32] detected two to six phases corresponding with the accuracy of detection 0.2 < G < 0.6 based on HMM with the help of two IMUs mounted to the foot and shank. Evans and Arvind [28] proposed a sensor system equipped with seven IMUs placed on both sides of the feet, shanks, and thighs, and one on the pelvis combining with a hybrid method. This method was based on a Feed-Forward Neural Network (FNN) combining with a HMM. This system was tested on healthy adults with four working trials for identifying four or five gait phases in 20 ms or 23 ms, respectively. A robust gait phase detection method to identify the seven gait phases for walking normal gaits and walking pathologic gaits was introduced in study [15]. They estimated knee angles and tibia angles of the right leg in the sagittal using all of IMU signals. They wore four IMUs on the thigh, shank, and feet, and walked on the ground in a straight line with natural speeds. The algorithm based on an adaptive threshold in adaptive searching intervals to classify the key events in gait cycles. The detection results showed 100% accuracy for both normal and pathologic gaits. Later, Vu et al. [41] proposed a method that used all signals from one IMU placed on the shank. This study was capable of precisely predicting 100 percent of the gait cycle with the MSE achieving an average of 0.003 in both training and validation data sets. To sum up, the combination of angular velocity and linear acceleration with three inertial quantities produced a better performance such as improving the detection accuracy and decreasing the timing delay for the discrimination between phases.

#### 4.1.3. Measurements Based on Combination of IMUs and Force Sensors

The combination of inertial sensors with footswitches and foot pressure insoles outputs improves the robustness of the gait detection algorithms [23,34,46,82,83,84]. Gorsic et al. [23] showed an algorithm based on the principle of detecting transitions between gait phases. Signals for the algorithm were taken from seven IMUs attached to the body segments with signals from two pressure insoles. Results presented a highly accurate detection, on average 97% for four phases online. The detecting performance was similar with the HMM method while the algorithm was simpler without learning dataset acquisition and pre-model training. Senanayake et al. [83] proposed the concept of a fuzzy logic system to detect distinctly seven gait events. This algorithm was based on a real-time data acquisition system using four FSRs and two IMUs. The data inputs of this fuzzy logic system were taken from typical characteristic features of both the on/off status of FSRs during sub-stance phases and high/low peaks in knee flexion/extension angle of IMUs during sub-swing phases. The algorithm was tested on normal walking trials with errors in timing delay detection under 70 ms. A gait phase classification method with a NN-based method that took signal inputs from two IMUs and two to four FSRs was studied in [84]. The acceleration and gyroscope signals of lower limb segments were retrieved from the sensors attached to the lower limb exoskeleton ROBIN- H1, and data were fed into neural network-based gait phase classifiers. The offline results showed an excellent accuracy with an average of 97%, a better performance than online results. Lee et al. [46] implemented a smart insole consisting of a pressure sensor array, an acceleration sensor array, and a gyro sensor array based on deep CNN. This method determined the swing phase and stance phase for the main proposal of classifying the different types of gait.

These papers showed that the combination of IMUs and pressure insoles and foot switches improved algorithms of gait phase detection. More sub-phases can be recognized: Sub-stance phases detected by typical characteristics of pressure insole and footswitch outputs; sub-swing phases detected by typical features of IMUs are negative and positive peaks of angular velocity or linear acceleration outputs. The algorithms in these systems were basically approached by the means of HMM, threshold method, and a fuzzy inference system.

#### 4.1.4. Measurements Based on EMG Signals

The use of EMG sensors cover surface measures specific to muscle activities occurring during movements. Several studies took advantage of the surface (EMG) signal to recognize the gait event system [36,56,85,86,87]. However, EMG-based systems are less commonly used for gait detection because they have poor performance in clinical trials due toelectrode position changes, electrode shift, the effects of nerve atrophy, and the muscle variations. The combinations of two algorithms, ANN and LDA to identify two events, HC and TO, obtained a 85.7% of detection accuracy [88]. One model based on Support Vector Machines (SVMs) proposed a great result with over 95% of accurate five phases detected [89]. Joshi el al. [56] proposed a control system based on LDA that could detect eight gait phases by using eight EMG signal outputs placed under the feet of their foot-knee exoskeleton. However, the signal processing computation was complicated with mean absolute value, waveform length, variance, and slope sign change. The EMG-based was less popular because of the complex requirements in data acquisition and processing steps, the sensitivity generated by moisture that builds between the skin and sensors, as well as the way in which they are placed under the skin of the person.

### 4.2. Computation Methods

The computation methods of gait detection can be divided into several main categories. The simplest gait phase detection methods are based on the identification of peaks and threshold rules. More elaborate methods are machine learning approaches such as HMMs, neural networks, and classification by Fisher’s linear discriminant.

**Figure 3:**
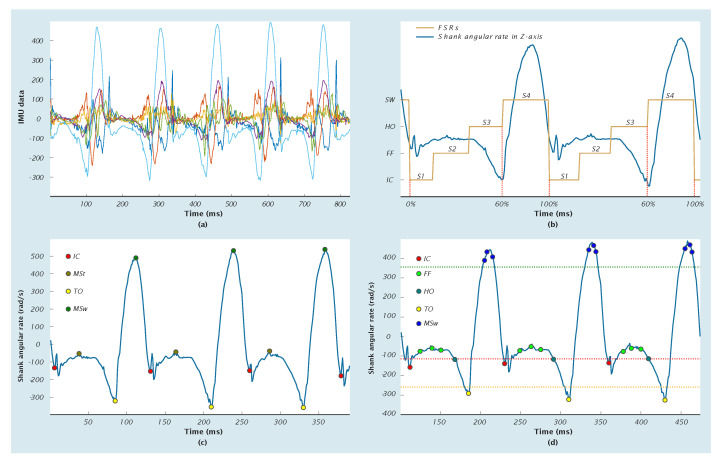
(**a**) Shank acceleration signals and angular velocity signals for the training model of Artificial Neural Network technique (ANN) methods, (**b**) using Force Sensing Resistors (FSRs) data for event detection and for labeling data input of the Hidden Markov Models (HMM) technique, (**c**) the identification of peaks for gait event detection, and (**d**) a proposal of the base-line threshold for the phase and event detection.

Figure 3 illustrates several methods based on the general features of IMU raw signals and FSRs signals. For instance, Figure 3a shows the acceleration and angular velocity signals of the shank used as the input training data [38,41]. Four phases of the IC, FF, HO, and TO could be obtained by using two FSRs placed under the heel and toe as shown in Figure 3b. The data of two FSRs also marked four phases for the training label data for HMM approaches to detect possible transitions among four states of S1, S2, S3, and S4 [29,30]. Figure 3c shows several peaks of angular velocity signals at the shank corresponding gait phases. The first negative peak marked IC and FF during the period that the angular rate was near to zero. Mid-stance (MSt) was possibly detected by the peak closest to zero during the FF. The negative peak after the FF was detected as the TO. The mid-swing (MSw) phase is marked by the maximum magnitude of the angular velocity signal c [16,19,77]. Figure 3d describes several threshold rules associated with gait phases such as the LR, FF, SW, and ST [17,90]. In addition, it was possible to calculate the gait phases by adaptive threshold lines for locating the peaks of the shank angular rate [91].

#### 4.2.1. Threshold Methods

The simplest computation methods of the gait detection are the threshold values. There are different types of threshold algorithms as a set of value rules that figures out certain characteristics of the gait phases or events [9,10,11,12,13,15,90], time-frequency analysis method based on thresholding values [18,19,20,21,92], or a peak heuristic algorithms which is also a branch of the threshold method in case the derivative passes through zero [16,19,23,77,93]. From the sensor signals, the shank angular velocity signal of the sagittal plane shows clearly two negative peaks at the IC and FO events, this is the simple rule to determine IC and FO events with a high accuracy of detection [11,19,79,82]. Detection success was over 93% [79] and over 98% [11]. The IC and FO events of walking in two terrains were detected in overall reliability 95% for the TO, 99% for the IC in the upstairs scenario, 99% for the TO, and 98% for the IC in the downstairs scenario. Mariani et al. [25] proposed a method to detect a common stance phase and stance phase, as well as sub-stance phases that are loading response, foot-flat, and push-off using one IMU placed on the foot. The peak method-based was used to detect the phases. This algorithm showed the reliable precision of detection that could be further implemented in real-time for applications.

Two studies [15,94] implemented a similar method as the complex threshold rules that were further applied in exoskeletons. The method of Meng at al. [15] could detect seven gait phases by the estimation of knee angles and tibia angles in the sagittal of the right leg from all IMU signals with a great detection result of 100% reliability. Boutaayamou et al. [94] proposed a method that could detect four events with a temporal accuracy of around 10 milliseconds. Both systems [15,94] required four sensors that were placed on the segments of the legs. Real-time validation of the event detection systems thresholding-based showed significant delays. For example, the combination of heuristics and zero-crossing method to determine HS and TO recorded the latency at an average of 100 ms [77]. Maqbool et al. [95] recorded the time delays of their proposed method 21.8±20 ms (IC), −7.5±15.5 ms (TO), and −1.7±53 ms (HO). After that, Maqbool and his colleagues proposed an algorithm based on heuristic rules that obtained a high performance of detection compared to their existing study [9]. The mean difference error between the proposed and reference system was about +4 ms (HC) and about −6.5 ms (TO) [16]. Gait phase and event detection systems that are threshold based are simple and reliable to implement in applications that require two detected events in the TO and IC. Nevertheless, these detection systems could not be useful for applications in terms of sub-phases requirement.

#### 4.2.2. Machine Learning Methods

ML methods are among the most popular techniques used to classify gait phases in off-line data as well as in real-time data. Many gait phase recognition methods have been developed using different types of machine learning approaches such as HMM, NN model, DLNN, and CNN. For instance, HMM, a branch of ML was proposed in various applications [26,27,28,29,30,32,33,34,84,96]. This technique always detects four event phases [27,29,30] for both online detection [28,30] or offline detection [26,27,29]. Mannini et. al [30] applied HMM to detect four gait phases HS, FF, HO, and TO real-time using the foot angular velocity in the sagittal plane measured by a uniaxial gyro placed on the foot. This study showed a delay limitation in online detection with an average latency of 62 ± 47 ms for FS and 86 ± 61 ms for HO. The method presented in [96] used the kinematic features of the gait signals as inputs. The accuracy of phase detection was higher than 94%, with a latency of nearly 35 ms. Sanchez et al. [29] proposed a method based on HMM to detect four phases of HS, FF, HO, and SP. However, there is no potential for online computation since the dimensions of the generated parameters were too big, therefore the latency will be a problem when conducting heavy mathematical computations. There was a study that proposed a gait system that exhibited the ability to detect from two to six phases corresponding with the accuracy 0.2 < G < 0.6 based on HMM with the help of two IMUs mounted to the shank and foot [32]. Before that, Bae et al. [26] identified six gait phases using the output signals from four ground reaction forces. The result showed the classification of abnormal and normal gait phases, and the application was the potential of diagnosing the improvement of a patient’s rehabilitation treatment without regard to the accuracy of detected phases.

Many methods have been developed based on an artificial neural network to estimate parameters of the gait phase [28,60,84,97]. Liu et al. [60] presented a neural network model that could detect eight phases offline with an accuracy of detection that was only 87.2–94.5%. Researchers [84] also used two neural networks for gait phase classifiers showing excellent offline results with an average error rate of 97%, but the online performance was not reliable. Later, Evans and Arvind [28] boosted the number of detected event-phases to five based on a hybrid method that was combined with a Feed-Forward Neural Network (FNN) and a HMM. However, their sensor system used seven IMUs attached to body segments in the feet, shank, both sides of the thigh, and one on the pelvis, and the average detection accuracy of five phases calculated was 88.7% within 23 milliseconds in terms of a data capture rate of 42.67 frames per second. Related to labeling, the study analyzed IMU data within two phases to feed a CNN-based method for motion recognition [45]. In another study embedded a three layer NNs into a HMM for classification of six gait phases, and employed a rule-based detection method to label the training data [33]. This method obtained precise results of 98.11%, 94.32%, and 98.86% using metrics of accuracy, sensitivity, and specificity, respectively. This hybrid method was computationally complicated for model training but computationally efficient in real-time detection. There were two new studies that utilized deep learning models for their gait phase detection systems. The LSTM-DNN algorithm for gait detection to detect two main phases had a limitation accuracy of 91.8% by installing three sensors on the foot, thigh, and calf [73]. Another novel approach was presented to estimate the percent of the gait cycle based on DLNN using inertial sensor data for both tri-axis acceleration and tri-axis angular velocity mounted to the shank. This study reported a MSE of 0.003 in both training and validation datasets [41].

ML-based methods were overall an effective approach method for the gait phase detection with more sub-phases than other methods having eight phases [89] with even a 100 gait percent [41,64]. Many studies showed excellent offline results, however real-time detection was not remarkable since the computation costs time as the matrix sizes of parameters were too large [60,73,84].

## 5. Performance Comparison of Gait Detection Methods with Their Systems

Table 1 summarize the performance of different methods based on the measurement of common parameters as the detected granularity, and sensors, as well as the sensor placements, methods, and performance. There are many metrics that researchers can use in order to evaluate the performance of detection algorithms. Different gait phase detection systems are adopted to the metric differently depending on the algorithm.

- *Time difference*: The time distance between the temporal phase/event detected the reference as footswitches, force sensors, or vicon-system. The phase can be detected before or after the reference;
- *Detection delay*: The time cost of algorithm computation to obtain the output;
- *Detection precision*: Accurate percentage or error percentage. These metrics are counted by true detection phase/event or fail detection percent;
- *Goodness (G)*: The significant differences between the result and standardized observed data;
- *Detection rate*: Are defined to be sensitivity (true positive rate) and specificity (true negative rate).

These studies developed real-time algorithms [18,22,29,30,95,98] in which Mannini et al. [30] developed a HMM to detect four phases online used the gyroscope signals from four IMUs placed on the waist, thigh, shank, and foot, however the time difference of detection was too delay as 62 ± 47 ms (IC), −3 ± 53 ms (FF), 86 ± 61 ms (HO), and 36 ± 18 ms (TO). Another study from Muller et al. [98] also detected four phases online, and the time differences were even worse than study [30]: 100 ± 50 ms (TO) and 50 ± 79 ms (IC). Lee et al. [22] achieved a better result with a delay of 19 ms (IC) and −8 ms (TO), however this study could only detect two events for the IC and TO. In another article [18], Ledoux et al. introduced a method that could detect two events for the IC and TO, with a mean detection accuracy of 1.8±0.6% stride (TO) and 1.7±0.6% stride (IC). Among the offline methods, most applied ML models were HMMs [29,32], ANN [60], and deep learning [41,73].

Sanchez et al. [29] achieved a higher performance of detection compared to the methods presented in [30,32]. This detection method also based on the HMM used gyroscope signals from one IMU attached to the foot. Four gait phases were detected with the latency of less than 29±12 ms for the prosthetic group.

Adding more sensors attached to the body segments possibly detected more events and phases. For instance, Liu et al. [60] built the model based on ANN to achieve eight phases detection. The data for training this network was taken from four angle sensors placed on four joints of knees and hips. The recognition accuracy was 94% (CRS) and 84% (CRP). However, network computation costs time, compromising its use for real-time applications. Installing three sensors on the foot, thigh, and calf to feed the model LSTM-DNN was introduced in [73]. However, the performance of this model was not reliable with the accuracy limited to 91.8% and the model detecting two main phases.

We observed that there were two papers where systems provided phase detection discretized within a 1% interval. Quintero et al. [64] estimated the gait phase variable percent by calculating the angles and rates by the linear relationship in the polar coordinate. However, this study proposed offline analysis without investigating the performance of the phase detection method. Later, the algorithm called Exponentially Delayed Fully Connected Neural Network (ED-FNN) was presented to be capable to precisely predict 100 percent of the gait cycle and used the shank IMU signals. To our best knowledge, this setting has never been realized in previous studies. Furthermore, the results showed that the MSE achieved 0.003 in both validation and training datasets. Though this model was learned offline, the real-time detection performance was expected to be similar to the offline performance because the sizes of parameters were reduced in their method.

## 6. Conclusions

In this survey, a large number of studies related the state-of-the-art gait phase detection was screened. The review provided a useful summary of current research and sheds light on potential future research directions. We realized that phase and event detection systems potentially employed IMU sensors since they were the best suited for long-term applications in their daily activities. IMUs overcame the limitations of force sensors in terms of energy consumption, durability, cost, weight, portability, and ease of placement. Additionally, signals from IMUs could be used for all gait phase detection methods mentioned in Section 4. On the other hand, the force sensors could address the reference for the experiment and validation of the system. Detection of two phases could be addressed by every method with an accuracy to 100%. The ML could tackle the detection of sub-phases from four to eight phases even more. If the granularity was required to be increased, this would lead to a decrease in accuracy in the same gait detection system. This issue could be addressed by using hybrid algorithms or using complex algorithms and more parameters involved, which also showed a greater result. In addition, signal post-processing to avoid errors, drift, and noise of the signals was one of the best ways to obtain a high performance. However, adding more calculation steps could cause delay, in particular the detection delay was important in real-time applications. In summary, it depends on the prosthetic design requirements to consider the number of detected proper granularity while improving the detection accuracy and decreasing the detection latency.

## Figures and Tables

**Table 1:** Existing gait phase and event detection methods applied on lower limbs.

Authors	Sensor Types	Placements	Methods	Detectable Events or Phases	Performance	Metrics	Detections
Zhen et al. [73] (2019)	Three IMUs	Foot, thigh, and calf	LSTM-DNN	Two phases	91.8% (Accuracy) 92% (F-score)	Detection precision	Off-line
Liu et al. [60] (2016)	Four angular sensors	hips, knees	NN	8 phases	94.5% (CRS) 87.22% (CRP)	Detection precision	Off-line
Sanchez et al. [29] (2019)	One IMU	Foot	HMM, TB	HS, FF, HO, SP	Prosthetic Group −17±20 ms (IC), TP Method −28±12 ms (FF), 9±29 ms (HO), −24±15 ms (SP), −22±16 ms (IC), SST Method −29±12 ms (FF), −19±18 ms (HO), −27±14 ms (SP)	Time difference	Off-line
Ledoux et al. [18] (2018)	One IMU	Shank	THR, LDA, QDA	IC and TO	92%	Detection precision	On-line
Zakria et al. [16] (2017)	One IMU	Shank	Heuristicrule set	IC, TO, MSw and MSt	3.92±1.56 ms (IC), −1.81±4.03 ms (TO)	Time difference	Off-line
Maqbool et al. [95] (2016)	One IMU	Shank	Rule-based	IC, TO and HO	21.8±20 ms (IC) −7.5±15.5 ms (TO) −1.7±53 ms (HO)	Time difference	On-line
Zhou et al. [19] (2016)	One IMU	Shank	TF and HA	IC and TO	95% (TO: upstairs), 99% (IC: upstairs), 99% (TO: downstairs) 98% (IC: downstairs)	Detection precision	off-line
Mannini et al. [30] (2014)	Four IMUs	Waist, thigh, shank and foot	HMM	IC, FF, HO, TO	62±47 ms (IC), −3±53 ms (FF), 86±61 ms (HO), 36±18 ms (TO)	Time difference	On-line
Agostini et al. [47] (2014)	Three FSs	Under the sole	Binary coding	IC, FF, HO, TO	100% (Healthy subjects) 98% (Patients)	Detection precision	Off-line
Mannini & Sabatini [96] (2011)	One IMU	Foot	HMM	IC, FF, HO, TO	>94% 20 ms	Detection precision	Off-line
Bae et al. [26] (2011)	Four GRFs	Under the sole	HMM	Detected 6 phases	NoN	NoN	Off-line
Evans and Arvind [28] (2014)	Seven IMUs	Feet, shanks, thighs and pelvis	FNN, HMM	Detected five phases	88.7% 23 ms (IC)	Detection precision	On-line
Attal et al. [34] (2018)	Pressure	Feet	MRHMM	6 phases	83.21%	Detection rate	Off-line
Bejarano et al. [91] (2014)	2 IMUs	Shanks	THR	IC, TO, MSw	<31 ms	Detection delay	On-line
Taborri et al. [32] (2015)	Two IMUs	Shank, Foot	HMMs	2, 4 and 6 phases	0.02 < G < 0.6	Detection rate	Off-line
Zhao et al. [33] (2019)	Two IMUs	Feet	NN, HMM	6 phases	98.11% (Accuracy) 94.32% (Sensitivity) 98.86% (Specificity)	Detection rate	Off-line
Rueterbories et al. [12] (2014)	One IMU	Foot	Thresholds	LR, MS, PS, SW	84.2% (Healthy) 108.5% (Patient)	Detection rate	Off-line
Lee et al. [22] (2011)	One IMU	Foot	Peaks	IC, TO	19 ms (IC) −8 ms (TO)	Time difference	On-line
Quintero et al. [64] (2017)	IMU	Thigh	EM	100 gait percent	Reported visually	Theory	Off-line
Vu et al. [41] (2018)	One IMU	Shank	FNN	100 gait percent	2.1 ± 0.1%	MAE-No delay	Off-line
Kim et al. [21] (2020)	One IMU	Foot	Time-frequencyanalysis	IC, TO	97% (TO runing events) 99% (Other events)	Detection precision	Off-line
Yan et al. [57] (2020)	One IMU	Foot	THR	IC, TO	97% (TO runing events) 99% (Other events)	Detection precision	Off-line
